# Accelerated intracranial time-of-flight MR angiography with image-based deep learning image enhancement reduces scan times and improves image quality at 3-T and 1.5-T

**DOI:** 10.1007/s00234-025-03564-7

**Published:** 2025-03-17

**Authors:** Young Hun Jeon, Chanrim Park, Kyung Hoon Lee, Kyu Sung Choi, Ji Ye Lee, Inpyeong Hwang, Roh-Eul Yoo, Tae Jin Yun, Seung Hong Choi, Ji-Hoon Kim, Chul-Ho Sohn, Koung Mi Kang

**Affiliations:** 1 Seoul National University Hospital, Seoul, Republic of Korea; 2 Kangbuk Samsung Hospital, Seoul, Republic of Korea; 3 Seoul National University, Seoul, Republic of Korea

**Keywords:** Brain, Magnetic resonance angiography, Deep learning, Cerebrovascular disorders

## Abstract

**Purpose:**

Three-dimensional time-of-flight magnetic resonance angiography (TOF-MRA) is effective for cerebrovascular disease assessment, but clinical application is limited by long scan times and low spatial resolution. Recent advances in deep learning-based reconstruction have shown the potential to improve image quality and reduce scan times. This study aimed to evaluate the effectiveness of accelerated intracranial TOF-MRA using deep learning-based image enhancement (TOF-DL) compared to conventional TOF-MRA (TOF-Con) at both 3-T and 1.5-T.

**Materials and methods:**

In this retrospective study, patients who underwent both conventional and 40% accelerated TOF-MRA protocols on 1.5-T or 3-T scanners from July 2022 to March 2023 were included. A commercially available DL-based image enhancement algorithm was applied to the accelerated MRA. Quantitative image quality assessments included signal-to-noise ratio (SNR), contrast-to-noise ratio (CNR), contrast ratio (CR), and vessel sharpness (VS), while qualitative assessments were conducted using a five-point Likert scale. Cohen’s *d* was used to compare the quantitative image metrics, and a cumulative link mixed regression model analyzed the readers’ scores.

**Results:**

A total of 129 patients (mean age, 64 years ± 12 [SD], 99 at 3-T and 30 at 1.5-T) were included. TOF-DL showed significantly higher SNR, CNR, CR, and VS compared to TOF-Con (CNR = 183.89 vs. 45.58; CR = 0.63 vs. 0.59; VS = 0.73 vs. 0.61; all *p* < 0.001). The improvement in VS was more pronounced at 1.5-T (Cohen’s *d* = 2.39) compared to 3-T HR and routine (Cohen’s *d* = 0.83 and 0.75, respectively). TOF-DL also outperformed TOF-Con in qualitative image parameters, enhancing the visibility of small- and medium-sized vessels, regardless of the degree of resolution and field strength. TOF-DL showed comparable diagnostic accuracy (AUC: 0.77–0.85) to TOF-Con (AUC: 0.79–0.87) but had higher specificity for steno-occlusive lesions.

**CONCLUSIONS:**

Accelerated intracranial MRA with deep learning-based reconstruction reduces scan times by 40% and significantly enhances image quality over conventional TOF-MRA at both 3-T and 1.5-T.

**Supplementary Information:**

The online version contains supplementary material available at 10.1007/s00234-025-03564-7.

## Introduction

Three-dimensional (3D) Time-of-flight MR angiography (TOF-MRA) is a noninvasive, non-contrast vascular imaging technique that leverages flow-related enhancement [1]. This technique has been widely utilized in evaluating cerebrovascular diseases including intracranial aneurysm, steno-occlusive lesion, and arteriovenous malformation [2, 3]. Identifying intracranial vessel lesions is important as these lesions may lead to serious events such as aneurysmal subarachnoid hemorrhage (SAH), with a high fatality rate [4]. Therefore, early detection of vascular lesions on TOF-MRA may allow appropriate endovascular or surgical treatment, thereby decreasing mortality or morbidity.

Obtaining high-resolution images to assess small vascular structures or aneurysm morphology using 3D TOF-MRA can be time-consuming and often requires scan times exceeding 5 min [5–7]. By employing parallel imaging or compressed sensing techniques with higher acceleration factors, the scan time can be reduced, but the image quality is inadequate for clinical use [8, 9]. Moreover, previous studies have reported that TOF-MRA at 1.5-T offers a lower spatial resolution and may be more susceptible to image noise than 3-T [5, 10]. Nevertheless, a 1.5-T MR unit remains indispensable owing to improved patient safety and cost-effectiveness.

Recently, deep learning (DL)-based reconstruction of brain MRI, employing architectures, such as U-net and other deep neural networks (DNN), has emerged and has successfully generated high-spatial-resolution images with reduced scan time [11–13]. These models are trained on substantial image datasets during their training and internal validation phases, allowing them to produce high-resolution images from undersampled, low-resolution inputs. However, in prior studies on intracranial cerebrovascular imaging using DNN-based reconstruction, the focus was solely on achieving high-spatial-resolution images and reducing image noise, without any improvement in scan time [14, 15].

Therefore, this study aimed to assess the visualization of intracranial vessels using TOF-MRA with conventional and DL-based reconstruction and to further compare the image quality and diagnostic utility of vascular structures, considering variations in scan time and field strength.

## Materials and methods

### Study patients

**Fig. 1:**
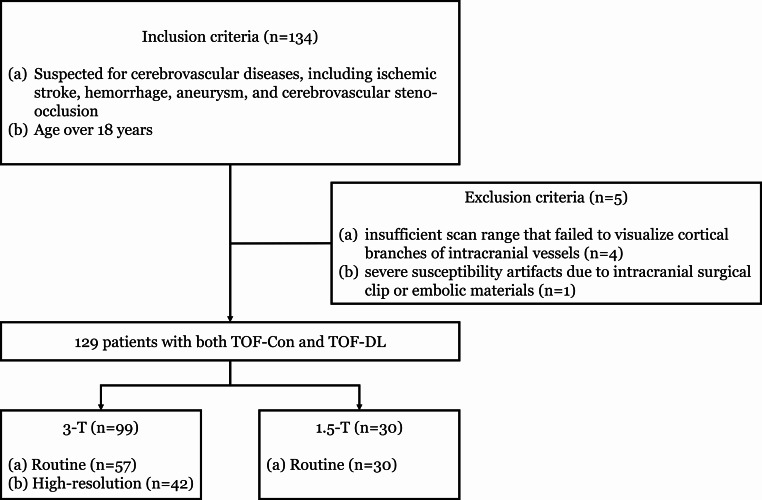
Flowchart shows the patient selection. Con = conventional, DL = deep learning

The Institutional Review Board of Seoul National University Hospital approved this retrospective study (IRB No. 2111-167-1276) and waived the requirement for written informed consent. We included consecutive patients who underwent TOF-MRA with baseline and accelerated scans from July 2022 to March 2023. These patients were included if they (a) were suspected of cerebrovascular diseases, including ischemic stroke, hemorrhage, aneurysm, and cerebrovascular steno-occlusion, (b) were older than 18 years of age. The exclusion criteria were as follows: (a) severe susceptibility artifacts due to intracranial surgical clip or embolic materials, and (b) insufficient scan range that failed to visualize cortical branches of intracranial vessels (Fig. 1).

### MR Acquisitions

**Fig. 2:**
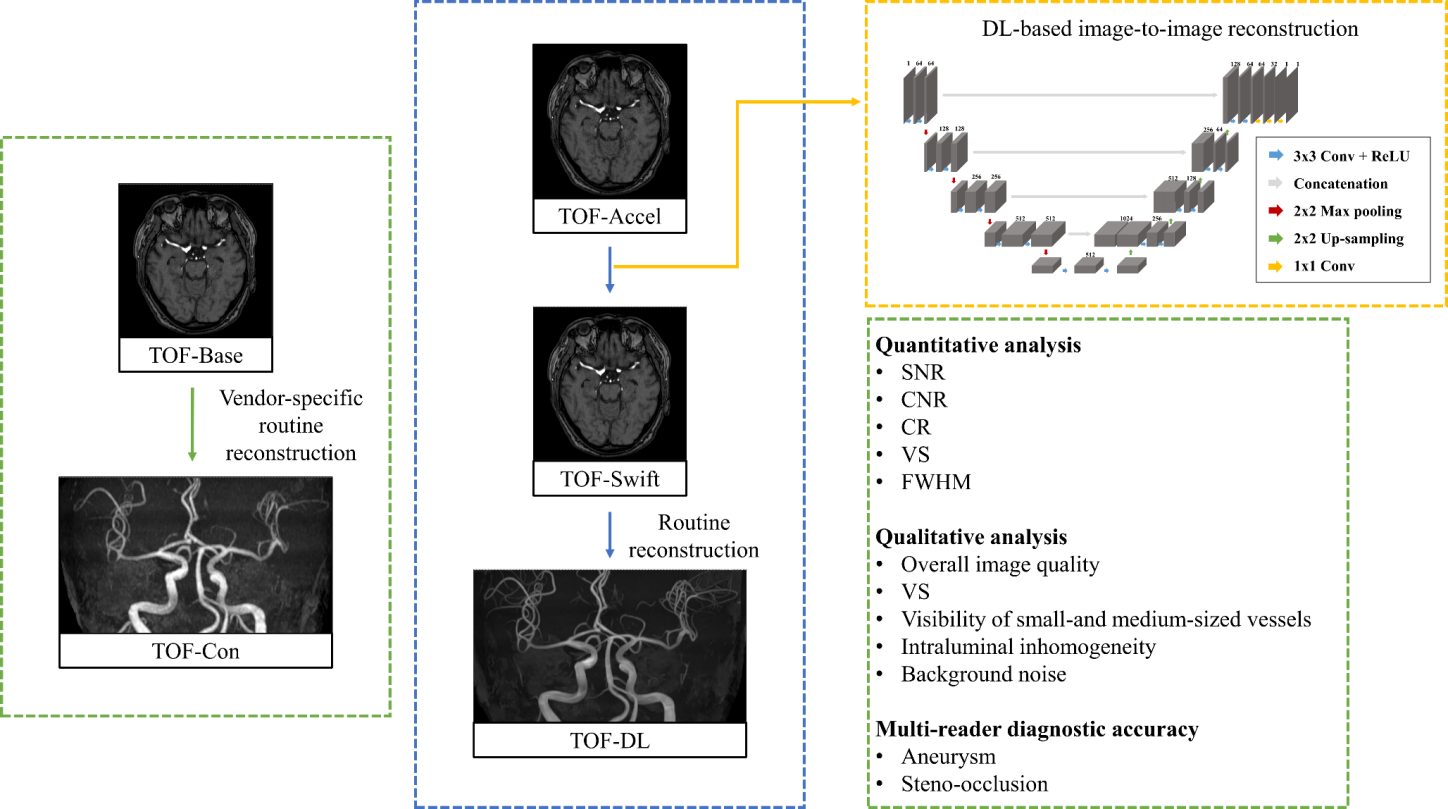
Schematic diagram of TOF-MRA acquisition with routine and deep-learning based image enhancement algorithm. Con = conventional, Accel = accelerated, DL = deep learning, CNR = contrast-to-noise ratio, CR = contrast ratio, VS = vessel sharpness, FWHM = full-width half maximum

MRI examinations were performed using either a 1.5-T scanner (Ingenia, Philips Healthcare, Best, Netherlands) or a 3-T scanner (Magnetom Skyra; Siemens AG Healthcare, Erlangen, Germany). All patients were imaged using two MRA sequences in the same session following a single protocol: baseline (TOF-Base) and accelerated scans (TOF-Accel). For the 3-T setting, protocols were categorized into routine- and high-resolution (HR) protocols. TOF-Accel reduced scan time by 40%, with acquisition times of 198 s (TOF-Base) and 122 s (TOF-Accel) for the routine protocol, and 624 s (TOF-Base) and 372 s (TOF-Accel) for HR protocol. At 1.5-T, only the routine protocol was applied, achieving the same 40% reduction, with acquisition times of 306 s (TOF-Base) and 186 s (TOF-Accel). Scan parameters are listed in Table S1 in the Supplementary Material. The maximum intensity projection (MIP) images of TOF-Base (TOF-Con) were automatically generated using the MR workstation’s 3D image processing software. A schematic diagram of MR acquisition is provided in Fig. 2.

### Deep learning-based image enhancement

This study utilized a commercially available, vendor-agnostic MR image enhancement software (SwiftMR, v2.0.1.0. AIRS Medical, Seoul, Korea). The model incorporates a DNN utilizing a 2D U-Net-based architecture [16] which is aimed to reduce image noise and improve the spatial resolution of images in the Digital Imaging and Communications in Medicine (DICOM) format. The foundational structure consists of convolutional block with 64 output channels including four down-sampling and up-sampling stages. Then the model is followed by 18 cascading convolutional blocks with down-sampling, up-sampling and feature concatenation layers, which sums up to three convolutional layers.

The software performs MR image denoising and resolution enhancement in a multi-dimensional manner enabled by training inputs generated from considerations such as undersampling patterns (uniform, random, k_max_, partial Fourier, elliptical, etc.) and noise amount in the image. The foundational U-Net architecture was modified to accommodate diverse image acquisition scenarios by enabling contextual data such as the acquisition parameters defining the k-space sampling as auxiliary input, resulting in a Context-Enhanced U-Net (CE U-Net) which incorporates a dynamic modulation pathway to utilize these contextual information from the input DICOM images.

Separate networks are used for 2D and 3D input images, where super-resolution is enabled in the direction of slice encoding for 3D images, on top of in-plane super-resolution. Detailed information regarding the software’s structure, training scheme, data used during the training and validation stages can be found in [17].

The algorithm was applied to the source image of TOF-Accel (TOF-Swift), then the corresponding MIP (TOF-DL) was created by transferring the post-processed source image back to the MR workstation and applying the identical MIP algorithm.

### Quantitative image quality analysis

A board-certified radiologist with 4 years of neuroimaging experience (Y.H.J.) quantitatively assessed the following imaging metrics: signal-to-noise ratio (SNR), apparent contrast-to-noise ratio (CNR), contrast ratio (CR), vessel sharpness (VS), and full-width at half maximum (FWHM) of the vessel diameter. SNR, CNR and CR were calculated from the source images using circular regions of interest (ROIs) at the mid-basilar artery (ROI_vessel_) and brainstem (ROI_tissue_) on the same slice. SNR was defined as the mean signal intensity (SI) of ROI_vessel_ divided by its standard deviation (SD). CNR was the mean SI of ROI_vessel_ divided by the SD of of ROI_tissue_. CR was calculated as the difference in mean SI between ROI_vessel_ and ROI_tissue_, normalized by their sum [18].

VS was assessed using a line profile perpendicular to the right middle cerebral artery (MCA), with FIJI’s “Line profile” function (ImageJ 1.46r Wayne Rasband, National Institute of Health, USA) [18, 19]; if the right MCA was not visible, the contralateral vessel was selected. Data from the line profile were normalized using the min-max normalization method, and VS was computed as the mean of the absolute slopes of the anterior and posterior vessel walls. The FWHM was determined from the same line profile curve, measured in pixels at half the maximum curve value [18] (Fig. S1 in the Supplementary Material).

### Qualitative image quality analysis

Four board-certified neuroradiologists (Y.H.J., K.H.L., J.Y.L., and K.S.C., with 4, 5, 11, and 6 years of experience, respectively), blinded to the clinical information and imaging technique, independently evaluated a couple of TOF-MRA MIP image sets in a randomized crossover manner, with at least four weeks of time interval between each review. The readers scored the qualitative image parameters using a 5-point Likert scale.

First, the readers scored the following parameters: overall image quality, impression of the VS, visibility of small- and medium-sized vessels, intraluminal inhomogeneity, and background noise. Background noise refers to venous or extracranial vascular signals and unsuppressed skull base fat. Second, the readers scored the visualization of the following vessel segments: internal carotid artery (ICA) horizontal and vertical petrous segments, ICA-C4 (cavernous segment), ICA-C5 (clinoid segment), ICA-C6 (ophthalmic segment), ICA-C7 (communicating segment), ophthalmic artery (OA), MCA (M1-3 segments), anterior cerebral artery (ACA, A1-3 segments), posterior communicating artery (PcomA), posterior cerebral artery (PCA, P1-3 segments), basilar artery (BA), superior cerebellar artery (SCA), vertebral artery (VA), and posterior inferior cerebellar artery (PICA) [18, 20]. Both right and left sides were graded together. The detailed scoring systems are shown in Table S2 in the Supplementary Material.

### Diagnostic test and reference standard

A consensus by two experienced neuroradiologists (K.S.C. and K.M.K., with 6 and 15 years of experience, respectively) served as the reference standard, based on TOF-MRA (including source images) with or without digital subtraction angiography and CT angiography. All available clinical information was utilized to enhance diagnostic accuracy.

Being blinded to imaging protocols and clinical information, after at least a four-week washout period, three readers (Reader 1: Y.H.J., Reader 2: K.H.L, and Reader 3: J.Y.L), independently evaluated the presence of intracranial aneurysms and steno-occlusion using two MIP sets (TOF-Con and TOF-DL).

### Statistical analysis

Quantitative image metrics were compared using paired t-tests or Wilcoxon signed-rank tests, with effect sizes quantified using Cohen’s *d* (small: 0.20–0.50; moderate: 0.51–0.80; large: > 0.80). A cumulative link mixed model was used to compare the qualitative image parameters [21], with the cluster effect from multiple scorings by the four reviewers on the same patient modeled as random effects. This model tested the impact of imaging protocol, scan time, and field strength, including a subgroup analysis at 1.5-T and 3-T. The inter-reader agreement was examined using Kendall’s coefficient of concordance (W). W coefficient values were interpreted as poor (κ ≤ 0), slight (0 < κ ≤ 0.20), fair (0.20 < κ ≤ 0.40), moderate (0.40 < κ ≤ 0.60), substantial (0.60 < κ ≤ 0.80), and almost perfect (0.80 < κ ≤ 1.00) [22]. The diagnostic performance of the readers was evaluated using receiver operating characteristic (ROC) curve analysis, and the areas under the curve (AUC) were compared using the DeLong test [23]. Sensitivity, specificity, and diagnostic accuracy were also calculated.

Statistical analyses were performed using R language version 4.2.2 (R Core Team, 2020). *p* values < 0.05 were considered statistically significant.

## Results

### Study patients

Of the 134 patients initially identified, 5 were excluded due to insufficient scan range (*n* = 4) or incomplete reading from severe susceptibility artifacts (*n* = 1). A total of 129 consecutive patients (mean age, 64 years ± 12 [SD]; 60 men) were finally included in the study. Among them, 99 underwent 3-T TOF-MRA (57 with routine and 42 with HR protocols), and 30 underwent 1.5-T TOF-MRA using the routine protocol. 63 patients (49.8%) exhibited at least one vascular pathology, with 40 presenting aneurysms and 32 showing steno-occlusions, while 66 patients (51.2%) displayed no vascular pathology (Table 1).

**Table 1:** Patient demographics

	3-T		1.5-T
Characteristic	Routine (*n* = 57)	HR (*n* = 42)	Routine (*n* = 30)
Patient age (y)*	65 ± 13	63 ± 10	66 ± 12
Patient sex			
No. of male	29	12	19
No. of female	28	30	11
Vascular pathology			
Aneurysm	7	29	4
Steno-occlusive lesion	18	4	10

Note.—Data are numbers of patients, unless otherwise noted. HR = high-resolution

* Data are means ± standard deviation

### Quantitative image analysis

**Fig. 3:**
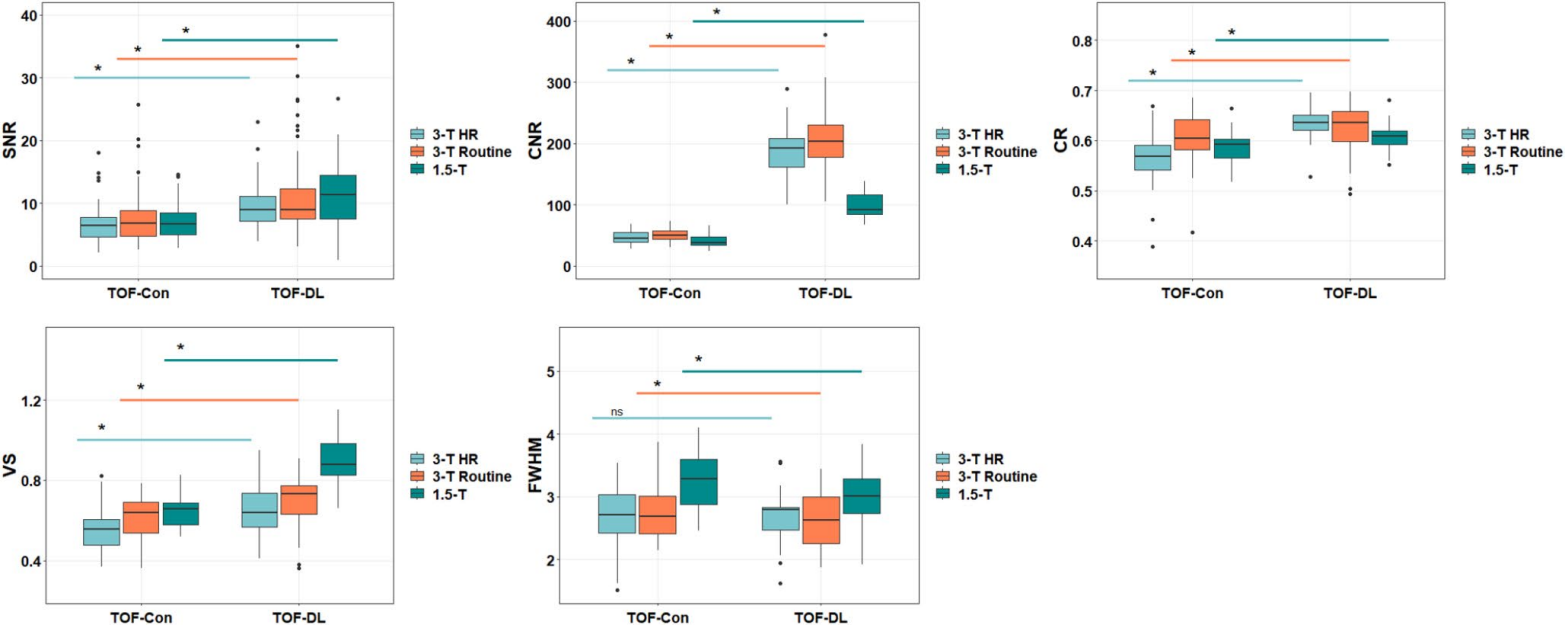
The box and whisker plots show the comparison of five quantitative image metrics between conventional (TOF-Con) and DL-based image enhanced time-of-flight MR angiograms (TOF-DL) according to field strengths and degree of resolution. Con = conventional, DL = deep learning, HR = high-resolution, SNR = signal-to-noise ratio CNR = contrast-to-noise ratio, CR = contrast ratio, VS = vessel sharpness, FWHM = full-width half maximum, ns = not significant

TOF-DL showed significantly higher SNR, CNR, CR, and VS values than TOF-Con (SNR: 9.24 vs. 6.74; CNR: 183.89 vs. 45.58; CR: 0.63 vs. 0.59; VS: 0.73 vs. 0.61; all *P* < 0.001), but a lower FWHM value (2.71 vs. 2.81, *P* = 0.001) (Table 2; Fig. 3). These trends persisted across the field strength subgroups, although no difference was noted for the FWHM within the 3-T HR subgroup (*P* = 0.73) (Table S3 in the Supplementary Material).

**Table 2:** Comparison of quantitative Image Metrics between TOF-DL and TOF-Con

Parameter	TOF-DL	TOF-Con	*P*	Cohen’s d^*^
SNR	9.24 [7.52, 12.80]	6.74 [4.71, 8.36]	< 0.001^†^	0.73 (0.50, 0.91)
CNR	183.89 [129.85, 208.35]	45.58 [44.20, 48.62]	< 0.001^†^	2.29 (1.98, 2.60)
CR	0.63 [0.60, 0.65]	0.59 [0.56, 0.62]	< 0.001^†^	0.83 (0.65, 1.01)
VS	0.73 [0.63, 0.83]	0.61 [0.53, 0.68]	< 0.001^†^	0.92 (0.76, 1.09)
FWHM	2.71 ± 0.42	2.81 ± 0.51	0.001	-0.20 (-0.31, -0.08)

Note.— Data are reported as means ± standard deviations when normally distributed and medians with interquartile ranges when the distribution is skewed. P values were obtained by using the paired *t* test or Wilcoxon signed-rank test, as appropriated. DL = deep learning, Con = conventional, SNR = signal-to-noise ratio, CNR = contrast-to-noise ratio, CR = contrast ratio, VS = vessel sharpness, FWHM = full-width at half maximum

^*^Number in parentheses are 95% confidence intervals. ^†^Compared using Wilcoxon signed rank test

Subgroup analysis revealed that CNR and CR had larger effect sizes at 3-T HR (Cohen’s *d* = 5.25 and 1.64) than 3-T routine (Cohen’s *d* = 4.34 and 0.50) and 1.5-T (Cohen’s *d* = 2.85 and 0.59) subgroups, However, SNR and VS demonstrated a greater effect size at 1.5-T (Cohen’s *d* = 0.98 and 2.39) than at 3-T HR (Cohen’s *d* = 0.75 and 0.83) or routine (Cohen’s *d* = 0.63 and 0.75) subgroups.

### Image quality parameters

**Fig. 4:**
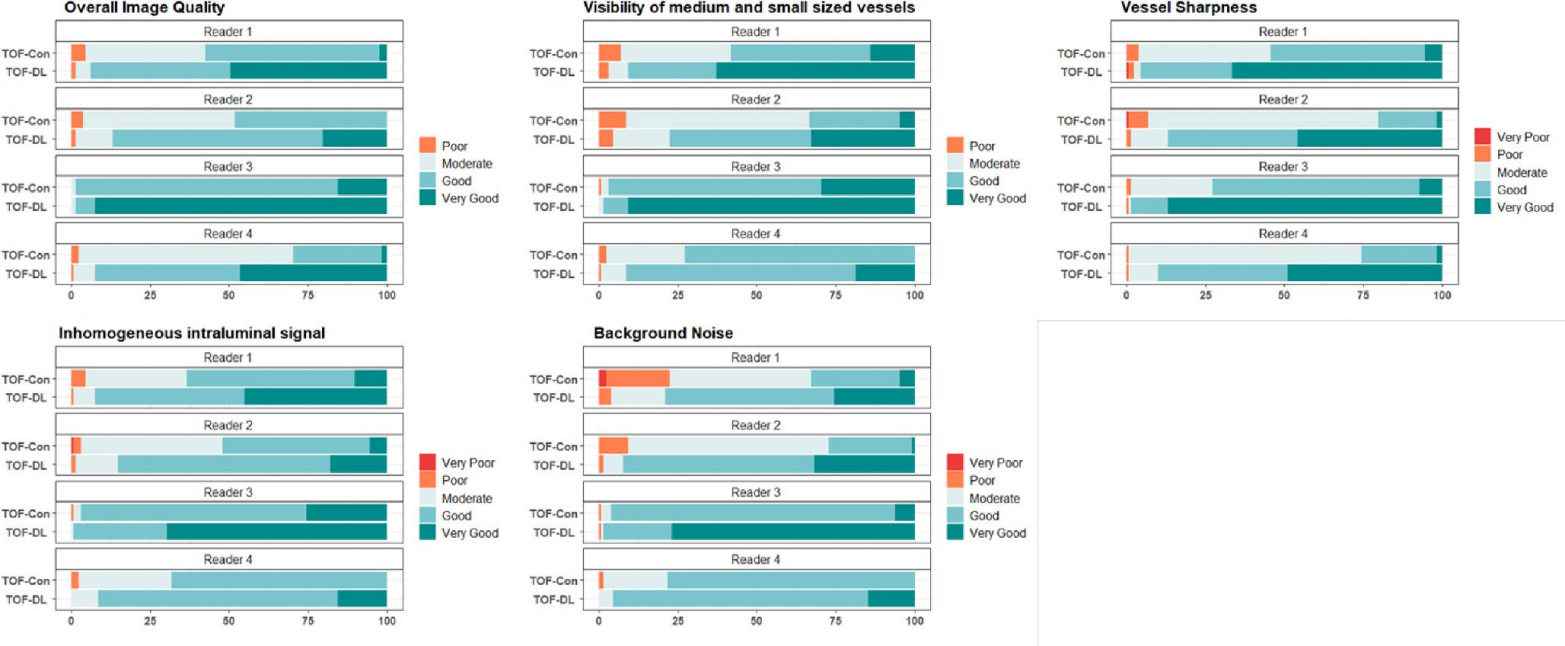
Bar graphs show the distributions of four reviewers’ scoring of the five qualitative image parameters on both conventional (TOF-Con) and deep learning-based reconstructed time-of-flight MR angiograms (TOF-DL). Con = conventional, DL = deep learning

As shown in Table 3; Fig. 4, for both 3- and 1.5-T, TOF-DL consistently showed a higher likelihood of superior scores across all categories than in TOF-Con, even after adjusting for field strength (all *P* < 0.001).

**Table 3:** Results of TOF-DL effect from cumulative link mixed model: qualitative image parameters

	All				3-T				1.5-T	
	Univariable		Multivariable^*^		Univariable		Multivariable^†^		Univariable	
Parameter	OR	*P* value	OR	*P* value	OR	*P* value	OR	*P* value	OR	*P* value
Image quality	49.66	< 0.001	49.66	< 0.001	36.73	< 0.001	36.70	< 0.001	198.60	< 0.001
VS	46.96	< 0.001	46.77	< 0.001	32.44	< 0.001	32.40	< 0.001	496.66	< 0.001
Visibility of small- and medium-sized vessels	15.02	< 0.001	15.03	< 0.001	10.46	< 0.001	10.48	< 0.001	69.53	< 0.001
Intraluminal inhomogeneity	8.53	< 0.001	8.55	< 0.001	5.96	< 0.001	5.96	< 0.001	35.23	< 0.001
Background noise	22.24	< 0.001	22.24	< 0.001	19.57	< 0.001	19.58	< 0.001	36.41	< 0.001

Note.— Cumulative link mixed model was used for univariable and multivariable analyses. DL = deep learning, VS = vessel sharpness

^*^ Adjusted for field strengths. Field strengths were considered as fixed effects, while multiple scorings by the four reviewers on the same patient were considered as random effects

^†^ Adjusted for degree of resolution. Degree of resolution was considered as fixed effects, while multiple scorings by the four reviewers on the same patient were considered as random effects

In the 3-T subgroup, TOF-DL outperformed TOF-Con across all image quality parameters, showing significant results in both the routine and HR protocols (all *P* < 0.001). Notably, the overall image quality and VS exhibited greater improvements in the routine protocol than in the HR protocol (OR: 104.01 vs. 14.12 for overall image quality and 60.55 vs. 17.27 for VS) (Table S4 in the Supplementary Material).

### Visibility of small- and medium-sized vessels

As shown in Table 4, TOF-DL significantly outperformed TOF-Con in depicting all vessel segments, even after adjusting for field strength and degree of resolution (all *P* < 0.001).

**Table 4:** Results of TOF-DL effect from cumulative link mixed model: visibility of small- and medium-sized vessels

	All				3-T				1.5-T	
	Univariable		Multivariable^*^		Univariable		Multivariable^†^		Univariable	
Segments	OR	*p* value	OR	*p* value	OR	*p* value	OR	*p* value	OR	*p* value
ICA_Ho	14.35	< 0.001	14.29	< 0.001	9.11	< 0.001	9.11	< 0.001	102.42	< 0.001
ICA_Ver	9.85	< 0.001	9.85	< 0.001	7.28	< 0.001	7.27	< 0.001	35.78	< 0.001
ICA_C4	9.85	< 0.001	11.6	< 0.001	7.4	< 0.001	7.4	< 0.001	105.16	< 0.001
ICA_C5	11.61	< 0.001	14.7	< 0.001	9.92	< 0.001	9.92	< 0.001	104.9	< 0.001
ICA_C6	14.7	< 0.001	16.21	< 0.001	11.78	< 0.001	11.78	< 0.001	55.43	< 0.001
ICA_C7	11.97	< 0.001	11.97	< 0.001	8.38	< 0.001	8.38	< 0.001	48.1	< 0.001
OA	2.78	< 0.001	2.79	< 0.001	2.01	< 0.001	2.01	< 0.001	13.37	< 0.001
PcomA	2.99	< 0.001	2.98	< 0.001	2.65	< 0.001	2.65	< 0.001	4.84	< 0.001
M1	8.27	< 0.001	8.29	< 0.001	4.91	< 0.001	4.91	< 0.001	91.13	< 0.001
M2	10.29	< 0.001	10.29	< 0.001	6.02	< 0.001	6.02	< 0.001	125.21	< 0.001
M3	17.59	< 0.001	17.59	< 0.001	12.19	< 0.001	12.19	< 0.001	80.62	< 0.001
A1	11.4	< 0.001	11.4	< 0.001	7.94	< 0.001	7.94	< 0.001	49.46	< 0.001
A2	4.2	< 0.001	4.2	< 0.001	2.84	< 0.001	2.84	< 0.001	25.63	< 0.001
A3	10.65	< 0.001	10.65	< 0.001	7.71	< 0.001	7.71	< 0.001	42.63	< 0.001
BA	12.07	< 0.001	11.97	< 0.001	10.28	< 0.001	10.28	< 0.001	21.59	< 0.001
SCA	8.50	< 0.001	8.52	< 0.001	6.86	< 0.001	6.86	< 0.001	23.06	< 0.001
P1	9.46	< 0.001	9.45	< 0.001	6.81	< 0.001	6.81	< 0.001	41.91	< 0.001
P2	10.15	< 0.001	10.11	< 0.001	7.28	< 0.001	7.28	< 0.001	48.1	< 0.001
P3	12.67	< 0.001	12.71	< 0.001	9.58	< 0.001	9.58	< 0.001	36.99	< 0.001
VA	10.9	< 0.001	10.88	< 0.001	7.72	< 0.001	7.72	< 0.001	36.36	< 0.001
PICA	5.59	< 0.001	5.59	< 0.001	5.35	< 0.001	5.35	< 0.001	6.5	< 0.001

Note.— DL = deep learning, ICA = internal carotid artery, ICA_Ho = ICA horizontal petrous segment, ICA_Ver = ICA vertical petrous segment, ICA_C4 = ICA cavernous segment, ICA_C5 = ICA clinoid segment, ICA_C6 = ICA ophthalmic segment, ICA_C7 = ICA communicating segment, OA = ophthalmic artery, M1-3 = M1-3 segments of middle cerebral artery, A1-3 = A1-3 segments of anterior cerebral artery, PcomA = posterior communicating artery, P1-3 = P1-3 segments of posterior cerebral artery, BA = basilar artery, SCA = superior cerebellar artery, VA = vertebral artery, PICA = posterior inferior cerebellar artery

^*^ Adjusted for field strengths and degree of resolution. Field strengths and degree of resolution were considered as fixed effects, while multiple scorings by the four reviewers on the same patient were considered as random effects

^†^ Adjusted for degree of resolution. Degree of resolution was considered as fixed effects, while multiple scorings by the four reviewers on the same patient were considered as random effects

In the 3-T subgroup, TOF-DL exhibited better visualization of intracranial vessels than TOF-Con. Remarkably, all small- and medium-sized vessels, except for OA (Cohen’s *d*: 0.47 vs. 1.06), exhibited greater improvement in vascular visualization in the routine protocol than in the HR protocol (Table S5 in the Supplementary Material).

### Agreement of Scoring

Inter-reader agreement for image quality parameters was fair to moderate, showing similar results between TOF-Con and TOF-DL (W; 0.38 to 0.52 for TOF-Con vs. 0.38 to 0.47 for TOF-DL). For the visibility of small and medium-sized vessels, inter-reader agreement was also fair to moderate and comparable between the two methods (W; 0.39 to 0.58 for TOF-Con vs. 0.42 to 0.52 for TOF-DL) (Table S6 in the Supplementary Material).

### Diagnostic performance of Vessel Pathology

Among the 129 patients, consensus reading identified 33 with steno-occlusion and 36 with aneurysms. In the identification of intracranial aneurysm, the diagnostic performance of all readers with TOF-DL was comparable to those with TOF-Con (AUC for Reader 1: 0.84 vs. 0.80, *p* = 0.36; for Reader 2: 0.82 vs. 0.80, *p* = 0.64; and for Reader 3: 0.70 vs. 0.77, *p* = 0.14) (Table S7 in the Supplementary Material).

**Fig. 5:**
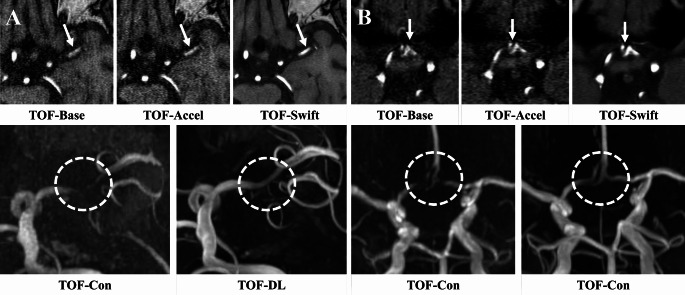
Zoomed-in areas of large-sized arteries on time-of-flight MR angiograms at 1.5-T. (**A**) A MIP image of conventional TOF-MRA (TOF-Con) shows a potential severe stenotic portion (dashed circle) in M1 segment of left middle cerebral artery (MCA) with markedly signal suppression on the source image (TOF-Base). DL-based image enhancement algorithm improves the visibility and pseudostenosis of left MCA in source (TOF-Swift) and MIP images (TOF-DL) compared to TOF-Con (dashed circles). (**B**) On MIP images of two TOF-MRA images, the degree of stenosis in both anterior cerebral arteries (dashed circles) appears to be more severe in TOF-Con than in TOF-DL. Con = conventional, DL = deep learning

**Fig. 6:**
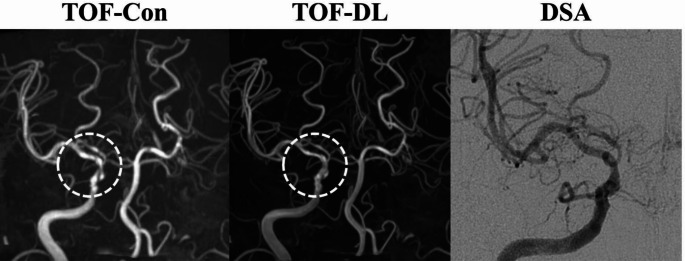
Intracranial TOF-MRA at 3-T. A MIP image of conventional TOF-MRA (TOF-Con) displays potential multiple stenotic portions (dashed circle) in the distal internal carotid artery (ICA) and M1 segment of right middle cerebral artery (MCA). A MIP image from the accelerated scans with DL-based image enhancement algorithm (TOF-DL) demonstrates reduced intraluminal signal inhomogeneity in the right distal ICA and MCA-M1, suggesting a false positive case of stenosis, which is confirmed by DSA. Con = conventional, DL = deep learning

In the identification of steno-occlusion, the diagnostic performance of all readers showed no significant differences between TOF-DL and TOF-Con (AUC for Reader 1: 0.93 vs. 0.86; Reader 2: 0.83 vs. 0.78; Reader 3: 0.85 vs. 0.84). Although the differences were not statistically significant, TOF-DL demonstrated higher specificity than TOF-Con in diagnosing steno-occlusive lesions, suggesting fewer false-positive results. Representative images are shown in Figs. 5 and 6.

## Discussion

In this study, we qualitatively and quantitatively evaluated a vendor-agnostic, DICOM-based DL image enhancement framework designed to accelerate TOF-MRA with a 40% reduction in scan time. Compared to conventional TOF-MRA, the DL framework demonstrated improved image quality, enhanced vascular visualization, and reduced background noise across various vendors and magnetic field strengths. Additionally, TOF-DL provided diagnostic accuracy comparable to TOF-Con in detecting cerebrovascular aneurysms and steno-occlusive disease.

A prior study by Wicaksono KP et al. demonstrated that MRA images reconstructed with a generalized adversarial network (GAN) surpassed low-resolution MRA in terms of image quality for both the distal and proximal segments of intracranial vessels while maintaining similar diagnostic efficacy in cerebrovascular disease assessment [14]. However, GAN-MRA did not outperform high-resolution MRA or reduce scan time. In contrast, our study showed that TOF-DL not only improved vessel visibility, overall image quality, VS, intraluminal inhomogeneity, and background noise compared to both routine and high-resolution TOF-Con but also reduced the scan time by 40%, enabling high-resolution MRA within approximately 2 min at 3T and 3 min at 1.5T. Quantitatively, TOF-DL outperformed TOF-Con in terms of SNR, CNR, CR, VS, and FWHM, consistent with our qualitative analysis.

A major limitation of TOF-Con is its tendency to overestimate stenosis due to flow-related enhancement [24, 25]. This overestimation is especially pronounced when the arterial flow is turbulent or parallel to the section plane, leading to signal loss from flow spin dephasing [26, 27]. Previous studies have attempted to mitigate these artifacts using ultrashort TE techniques [28, 29]. Our study applied DL-based image enhancement to generate high-resolution images from accelerated low-resolution scans. The U-Net architecture of this network improves vascular continuity by slice concatenation and differentiates vascular structures from noise [30], reducing false-positive results in the diagnosis of cerebrovascular steno-occlusive lesions.

DL-based image enhancement algorithms present certain challenges, particularly in preserving normal anatomical details. These algorithms may remove areas with significantly lower SNR than they were trained for, leading to over-smoothed images, especially with higher acceleration [17]. This can lead to undesirable issues such as the fusion of intracranial vessels and diminished visibility of cortical branches. In specific clinical conditions, these limitations may compromise diagnostic accuracy. For example, in patients with moyamoya disease, the loss of basal collaterals (Fig. S2 in the Supplementary Material) has been observed, while in cases of arteriovenous fistula, small feeders may become undetectable. To mitigate this issues, it is essential to utilize DL-based image enhancement algorithm at lower acceleration rates, as this approach is critical for preserving key diagnostic information. Additionally, since this method operates on DICOM data rather than raw k-space, it is likely less susceptible to Gaussian noise, thereby reducing the risk of image artifacts.

Our findings demonstrated that DL-based image enhancement improves image quality and vascular visualization, particularly at 1.5T, with higher effect sizes in VS. This improvement is likely attributable to the lower inherent signal, where the DL-based algorithm plays a more impactful role in recovering inherent signal and mitigating low spatial resolution artifacts, such as intravoxel dephasing, resulting in enhanced edge sharpness. These advancements may alleviate common diagnostic challenges at 1.5-T such as the overdiagnosis of steno-occlusive lesions [31, 32]. Moreover, our findings align with a previous study [15], suggesting that DL-based image enhancement effectively compensates for the lower SNR at 1.5-T.

3-T MRI scanners are frequently used to detect smaller aneurysms or remnants post-coil embolization [33–35]. To minimize susceptibility artifacts, strategies such as minimizing TE and increasing bandwidth are employed. While these adjustments effectively reduce artifacts, they may result in SNR reduction, which is often compensated for by increasing acquisition time. However, extended scan times can elevate the risk of motion artifacts, resulting in reduced image quality and potentially missed aneurysms, which could lead to severe outcomes including SAH [36, 37]. Our results revealed that even with high-resolution protocols, the acquisition time could be reduced by approximately 40%, while significantly improving image quality and visualization of small-sized vessels.

In a diagnostic test for intracranial vascular pathologies, no differences were found in the diagnosis of steno-occlusive diseases and aneurysms between TOF-Con and TOF-DL. These findings indicate that the DL-based image enhancement algorithm can provide comparable diagnostic performance in identifying cerebrovascular diseases while achieving an approximately 40% reduction in the acquisition time.

### Limitations

This study has several limitations. First, due to the single-center retrospective design, the diversity of patient demographics, types of MR vendors, and scan parameters were inherently limited. This limitation affects the generalizability of our findings. Second, only a subset of patients underwent DSA, the reference standard for cerebrovascular disease, which limits our ability to evaluate diagnostic performance for aneurysms, and steno-occlusive lesions. However, DSA is not routinely utilized in clinical practice for all cases due to its invasiveness, particularly in patients with small intracranial extradural aneurysms or mild cerebrovascular stenosis where aggressive intervention is not required. Third, our diagnostic tests did not evaluate vascular lesions other than steno-occlusions and aneurysms. Due to the limited number of positive cases, we were unable to further analyze other vascular pathologies, such as vasculitis, vascular malformations, and congenital vascular anomalies. Fourth, inter-reader agreement in the qualitative image evaluation was relatively low, likely reflecting variability in subjective assessments. To improve the generalizability and reliability of the findings, future studies with a multicenter design and a larger number of readers are recommended.

## Conclusion

Deep learning (DL)-based image enhancement algorithm significantly improves the efficiency of intracranial time-of-flight (TOF)-MRA with a 40% reduction in scan time, demonstrating superior image quality compared to conventional TOF-MRA at both 3-T and 1.5-T.

## Electronic supplementary material

Below is the link to the electronic supplementary material.

Supplementary Material 1

## Data Availability

No datasets were generated or analysed during the current study.

## Abbreviations

DL: Deep learning
Con: Conventional
HR: High-resolution
SNR: Signal-to-noise ratio
CNR: Contrast-to-noise ratio
CR: Contrast ratio
VS: Vessel sharpness
FWHM: Full-width at half maximum

## References

[1] Wheaton AJ, Miyazaki M (2012) Non-contrast enhanced MR angiography: physical principles. J Magn Reson Imaging 36:286–304 22807222 10.1002/jmri.23641

[2] MacDonald ME, Frayne R (2015) Cerebrovascular MRI: a review of state-of-the-art approaches, methods and techniques. NMR Biomed 28:767–791 26010775 10.1002/nbm.3322

[3] Ozsarlak O, Van Goethem JW, Maes M, Parizel PM (2004) MR angiography of the intracranial vessels: technical aspects and clinical applications. Neuroradiology 46:955–972 15580489 10.1007/s00234-004-1297-9

[4] Lawton MT, Vates GE (2017) Subarachnoid hemorrhage. N Engl J Med 377:257–266 28723321 10.1056/NEJMcp1605827

[5] Willinek WA, Born M, Simon B, Tschampa HJ, Krautmacher C, Gieseke J et al (2003) Time-of-Flight MR Angiography: comparison of 3.0-T imaging and 1.5-T imaging—initial experience. Radiology 229:913–920 14657322 10.1148/radiol.2293020782

[6] Lin Z, Zhang X, Guo L, Wang K, Jiang Y, Hu X et al (2019) Clinical feasibility study of 3D intracranial magnetic resonance angiography using compressed sensing. J Magn Reson Imaging 50:1843–1851 30980468 10.1002/jmri.26752

[7] Fushimi Y, Fujimoto K, Okada T, Yamamoto A, Tanaka T, Kikuchi T et al (2016) Compressed sensing 3-Dimensional Time-of-flight magnetic resonance angiography for cerebral aneurysms: optimization and evaluation. Invest Radiol 51:228–235 26606551 10.1097/RLI.0000000000000226

[8] Ding J, Duan Y, Zhuo Z, Yuan Y, Zhang G, Song Q et al (2021) Acceleration of Brain TOF-MRA with compressed sensitivity encoding: a Multicenter Clinical Study. AJNR Am J Neuroradiol 42:1208–1215 33858820 10.3174/ajnr.A7091 PMC8324268

[9] Tang H, Hu N, Yuan Y, Xia C, Liu X, Zuo P et al (2019) Accelerated time-of-flight magnetic resonance angiography with sparse Undersampling and Iterative Reconstruction for the evaluation of intracranial arteries. Korean J Radiol 20:265–274 30672166 10.3348/kjr.2017.0634 PMC6342758

[10] Kaufmann TJ, Huston J 3rd, Cloft HJ, Mandrekar J, Gray L, Bernstein MA et al (2010) A prospective trial of 3T and 1.5T time-of-flight and contrast-enhanced MR Angiography in the follow-up of coiled intracranial aneurysms. AJNR Am J Neuroradiol 31:912–918 20019107 10.3174/ajnr.A1932 PMC7964181

[11] Jung W, Kim J, Ko J, Jeong G, Kim HG (2022) Highly accelerated 3D MPRAGE using deep neural network-based reconstruction for brain imaging in children and young adults. Eur Radiol 32:5468–5479 35319078 10.1007/s00330-022-08687-6

[12] Koktzoglou I, Huang R, Ankenbrandt WJ, Walker MT, Edelman RR (2021) Super-resolution head and neck MRA using deep machine learning. Magn Reson Med 86:335–345 33619802 10.1002/mrm.28738 PMC8034362

[13] Muckley MJ, Riemenschneider B, Radmanesh A, Kim S, Jeong G, Ko J et al (2021) Results of the 2020 fastMRI Challenge for Machine Learning MR Image Reconstruction. IEEE Trans Med Imaging 40:2306–2317 33929957 10.1109/TMI.2021.3075856 PMC8428775

[14] Wicaksono KP, Fujimoto K, Fushimi Y, Sakata A, Okuchi S, Hinoda T et al (2023) Super-resolution application of generative adversarial network on brain time-of-flight MR angiography: image quality and diagnostic utility evaluation. Eur Radiol 33:936–946 36006430 10.1007/s00330-022-09103-9

[15] Yasaka K, Akai H, Sugawara H, Tajima T, Akahane M, Yoshioka N et al (2022) Impact of deep learning reconstruction on intracranial 1.5 T magnetic resonance angiography. Jpn J Radiol 40:476–483 34851499 10.1007/s11604-021-01225-2 PMC9068615

[16] Ronneberger O, Fischer P, Brox T (2015) U-Net: Convolutional Networks for Biomedical Image Segmentation. Med Image Comput Computer-Assisted Intervention Pt Iii 9351:234–241

[17] Jeong G, Kim H, Yang J, Jang K, Kim J (2024) All-in-One Deep Learning Framework for MR Image Reconstruction. *arXiv preprint arXiv:2405.03684*

[18] Sartoretti T, van Smoorenburg L, Sartoretti E, Schwenk A, Binkert CA, Kulcsar Z et al (2020) Ultrafast Intracranial Vessel Imaging with non-cartesian spiral 3-Dimensional Time-of-flight magnetic resonance angiography at 1.5 T: an in Vitro and clinical study in healthy volunteers. Invest Radiol 55:293–303 31895223 10.1097/RLI.0000000000000641

[19] Sartoretti T, Reischauer C, Sartoretti E, Binkert C, Najafi A, Sartoretti-Schefer S (2018) Common artefacts encountered on images acquired with combined compressed sensing and SENSE. Insights Imaging 9:1107–1115 30411279 10.1007/s13244-018-0668-4 PMC6269339

[20] Montalt-Tordera J, Quail M, Steeden JA, Muthurangu V (2021) Reducing contrast Agent Dose in Cardiovascular MR Angiography with Deep Learning. J Magn Reson Imaging 54:795–805 33619859 10.1002/jmri.27573 PMC9681557

[21] Bates D, Mächler M, Bolker B, Walker S (2015) Fitting Linear mixed-effects models Usinglme4. J Stat Softw: 67

[22] Landis J (1977) The measurement of Observer Agreement for Categorical Data. Biometrics 843571

[23] DeLong ER, DeLong DM, Clarke-Pearson DL (1988) Comparing the areas under two or more correlated receiver operating characteristic curves: a nonparametric approach. Biometrics: 837–845 3203132

[24] Tian X, Tian B, Shi Z, Wu X, Peng W, Zhang X et al (2021) Assessment of intracranial atherosclerotic plaques using 3D black-blood MRI: comparison with 3D time‐of‐flight MRA and DSA. J Magn Reson Imaging 53:469–478 32864816 10.1002/jmri.27341

[25] Stock K, Wetzel S, Kirsch E, Bongartz G, Steinbrich W, Radue E (1996) Anatomic evaluation of the circle of Willis: MR Angiography versus intraarterial digital subtraction angiography. Am J Neuroradiol 17:1495–1499 8883648 PMC8338732

[26] Yang JJ, Hill MD, Morrish WF, Hudon ME, Barber PA, Demchuk AM et al (2002) Comparison of pre-and postcontrast 3D time-of-flight MR angiography for the evaluation of distal intracranial branch occlusions in acute ischemic stroke. Am J Neuroradiol 23:557–567 11950644 PMC7975126

[27] Huang P, Chen K, Liu C, Zhen Z, Zhang R Visualizing Cerebral Small Vessel Degeneration during Aging and diseases using magnetic resonance imaging. *Journal of Magnetic Resonance Imaging*;n/a 10.1002/jmri.28736 37052571

[28] Fu Q, Zhang X-y, Deng X-b (2020) Liu D-x. Clinical evaluation of subtracted pointwise encoding time reduction with radial acquisition-based magnetic resonance angiography compared to 3D time-of-flight magnetic resonance angiography for improved flow dephasing at 3 Tesla. Magn Reson Imaging 73:104–110 32858182 10.1016/j.mri.2020.08.015

[29] Okuchi S, Fushimi Y, Okada T, Yamamoto A, Okada T, Hinoda T et al (2016) The pointwise encoding time reduction with radial acquisition (PETRA) sequence: visualization of intracranial arteries and facial nerve canals. In:*Proceedings of the 24th Annual Meeting and Exhibition of the International Society for Magnetic Resonance in Medicine*

[30] Jung W, Lee H-S, Seo M, Nam Y, Choi Y, Shin N-Y et al (2023) MR-self Noise2Noise: self-supervised deep learning–based image quality improvement of submillimeter resolution 3D MR images. Eur Radiol 33:2686–2698 36378250 10.1007/s00330-022-09243-y

[31] Shi Z, Zhao X, Zhu S, Miao X, Zhang Y, Han S et al (2023) Time-of-flight intracranial MRA at 3 T versus 5 T versus 7 T: visualization of distal small cerebral arteries. Radiology 306:207–217 36040333 10.1148/radiol.220114

[32] Runge VM, Heverhagen JT (2022) The clinical utility of magnetic resonance imaging according to field strength, specifically addressing the breadth of current state-of-the-art systems, which include 0.55 T, 1.5 T, 3 T, and 7 T. Invest Radiol 57:1–12 34510100 10.1097/RLI.0000000000000824

[33] Soize S, Gawlitza M, Raoult H, Pierot L (2016) Imaging Follow-Up of Intracranial aneurysms treated by Endovascular means: why, when, and how? Stroke 47:1407–1412 27026629 10.1161/STROKEAHA.115.011414

[34] Kapsalaki EZ, Rountas CD, Fountas KN The role of 3 Tesla MRA in the detection of intracranial aneurysms. Int J Vascular Med 2012;2012 10.1155/2012/792834 PMC3265088 22292121

[35] Pierot L, Portefaix C, Gauvrit JY, Boulin A (2012) Follow-up of coiled intracranial aneurysms: comparison of 3D time-of-flight MR angiography at 3T and 1.5T in a large prospective series. AJNR Am J Neuroradiol 33:2162–2166 22678846 10.3174/ajnr.A3124 PMC7965594

[36] Ji Y, Wu W, de Buck MHS, Okell T, Jezzard P (2023) Highly accelerated intracranial time-of-flight magnetic resonance angiography using wave-encoding. Magn Reson Med 90:432–443 37010811 10.1002/mrm.29647 PMC10953028

[37] Meixner CR, Liebig P, Speier P, Forman C, Hensel B, Schmidt M et al (2019) High resolution time-of-flight MR-angiography at 7 T exploiting VERSE saturation, compressed sensing and segmentation. Magn Reson Imaging 63:193–204 31434005 10.1016/j.mri.2019.08.014

