# Supplementary material for: Accelerated intracranial time-of-flight MR angiography with image-based deep learning image enhancement reduces scan times and improves image quality at 3-T and 1.5-T
Source: Neuroradiology. 2025 Mar 17;67(5):1203–13. doi: 10.1007/s00234-025-03564-7 (PMC12125137; doi:10.1007/s00234-025-03564-7)

**Supplementary Materials**

Table S1. Imaging Parameters of Time-of-flight MR Angiography Protocol according to Field Strengths

|  | 3-T | | | | 1.5-T | |
| --- | --- | --- | --- | --- | --- | --- |
|  | Baseline  routine | Accelerated  routine | Baseline high resolution | Accelerated high resolution | Baseline  routine | Accelerated routine |
| Repetition time (msec) | 22 | 22 | 23 | 23 | 27 | 27 |
| Echo time (msec) | 3.7 | 3.7 | 4 | 4 | 6.9 | 6.9 |
| Flip angle (degree) | 18 | 18 | 18 | 18 | 25 | 25 |
| Field of view (mm) | 220x180 | 220x180 | 207x207 | 207x207 | 210x210 | 210x210 |
| Acquisition voxel size (mm^3^) | 0.43x0.87x1.20 | 0.43x1.35x1.40 | 0.40x0.40x0.80 | 0.40x0.56x 0.88 | 0.55x0.65x1.20 | 0.41x0.87x1.50 |
| Matrix (frequency x phase) | 512 x 254 | 512 x 163 | 512 x 512 | 512 x 369 | 384 x 323 | 512 x 241 |
| Number of slices | 144 | 124 | 144 | 131 | 140 | 112 |
| Slice thickness (mm) | 0.6 | 0.7 | 0.4 | 0.44 | 0.6 | 0.75 |
| Acceleration factor | GRAPPA 2 | GRAPPA 2 | GRAPPA 2 | GRAPPA 2 | CS-SENSE 3 | CS-SENSE 3 |
| Number of slabs | 3 | 3 | 4 | 4 | 4 | 4 |
| Acquisition time (sec) | 198 | 122 | 624 | 372 | 306 | 186 |

Note.—GRAPPA=GeneRalized Autocalibrating Partial Parallel Acquisition, CS-SENSE=Compressed Sensing-Sensitivity Encoding

Table S2. The Detailed Scoring Systems of Qualitative Image Parameters

| **Score** | **Image quality** | **Vessel sharpness** | **Intraluminal inhomogeneity** | **Visibility of small- and medium-sized vessels** | **Background noise** |
| --- | --- | --- | --- | --- | --- |
| 5 | No artifacts, all vessel segments depicted with same quality, which is close to that as DSA | The display of vessels was excellent with sharp borders | Homogeneous intraluminal signal | Excellent depiction of vessel segment | Clear background noise without skullbase fat |
| 4 | Minor artifacts, all vessels in FOV clearly visualized but subtle reduced image quality | The display of vessels was good with clear borders | Subtle inhomogeneous intraluminal signal, but adequate for diagnosis | Good depiction of vessel segment and adequate for diagnosis | Mild background vascular noise or subtle unsuppressed skullbase fat, but adequate for pathology grade |
| 3 | Major artifacts, all vessels in FOV poorly visualized | The display of vessels was moderate with visible borders | Moderate inhomogeneous intraluminal signal, acceptable for diagnotic use with minor issues | Vessel segment visible, acceptable for diagnotic use with minor issues | Mild background vascular noise or subtle unsuppressed skullbase fat, acceptable for diagnostic use with minor issues |
| 2 | Severe artifacts and all vessels in FOV nearly nonvisualized | The display of vessels was poor with dim borders and affected the diagnosis | Markedly inhomogeneous intraluminal signal, insufficient evaluation for pathology grade | Vessel segment scarcely visible | Marked background vascular noise or extensive unsuppressed skullbase fat, insufficient evalution for pathology grade |
| 1 | All vessels in FOV invisible due to artifacts | The display of vessels was unclear and could not be diagnosed | Poor intraluminal signal and wall delineation, inadequate for diagnosis | Vessel segment invisible | Severe background vascular noise or extensive unsuppressed skullbase fat, inadequate for diagnosis |

Note.—FOV=field of view

Table S3. Comparison of Quantitative Image Metrics between TOF-DL and TOF-Con: Subgroup Analysis

|  | 3-T HR | | | | 3-T Routine | | | | 1.5-T | | | |
| --- | --- | --- | --- | --- | --- | --- | --- | --- | --- | --- | --- | --- |
| Parameter | TOF-DL | TOF-Con | *P* | Cohen’s *d^*^* | TOF-DL | TOF-Con | *P* | Cohen’s *d^*^* | TOF-DL | TOF-Con | *P* | Cohen’s *d^*^* |
| SNR | 9.59 ± 4.09 | 6.75 ± 3.51 | < 0.001 | 0.75 (0.40, 1.10) | 8.91 [7.52, 12.42] | 6.80 [4.79, 8.92] | < 0.001^†^ | 0.63 (0.25, 0.93) | 11.79 ± 5.70 | 7.31 ± 3.07 | < 0.001 | 0.98 (0.58, 1.36) |
| CNR | 191.78 ± 37.62 | 47.1 ± 10.33 | < 0.001 | 5.25 (4.14, 6.34) | 202.43 ± 48.33 | 51.13 ± 9.48 | < 0.001 | 4.34 (3.33, 5.19) | 99.03 ± 19.69 | 40.46 ± 9.73 | < 0.001 | 2.85 (2.31, 3.39) |
| CR | 0.63 ±0.03 | 0.56 ± 0.05 | < 0.001 | 1.64 (1.14, 2.11) | 0.63 ± 0.04 | 0.60 ± 0.05 | < 0.001 | 0.50 (0.29, 0.69) | 0.61 ± 0.03 | 0.59 ± 0.03 | < 0.001^†^ | 0.59 (0.27, 0.91) |
| VS | 0.66 ± 0.12 | 0.56 ± 0.10 | < 0.001 | 0.83 (0.54, 1.10) | 0.70 ± 0.12 | 0.61 ± 0.11 | < 0.001 | 0.75 (0.53, 1.0) | 0.89 ± 0.12 | 0.64 ± 0.07 | < 0.001 | 2.39 (1.62, 3.16) |
| FWHM | 2.68 ± 0.39 | 2.66 ± 0.54 | 0.73 | 0.04 (-0.2, 0.29) | 2.61 ± 0.40 | 2.72 ± 0.43 | < 0.001 | -0.28 (0.44, 0.14) | 2.96 ± 0.42 | 3.17 ± 0.43 | 0.001 | -0.49 (-0.78, -0.21) |

Note.— Data are reported as means ± standard deviations when normally distributed and medians with interquartile ranges when the distribution is skewed. P values were obtained by using the paired *t* test or Wilcoxon signed-rank test, as appropriated. HR=high-resolution, DL=deep learning, Con=conventional, SNR=signal-to-noise ratio, CNR=contrast-to-noise ratio, CR=contrast ratio, VS=vessel sharpness, FWHM=full-width at half maximum

^*^Number in parentheses are 95% confidence intervals. ^†^Compared using Wilcoxon signed rank test

Table S4. Results from Cumulative Link Mixed Model Analysis: Resolution Degree at 3-T Using Qualitative Image Parameters.

|  | Routine | | HR | |
| --- | --- | --- | --- | --- |
| Parameter | OR | *P* value | OR | *P* value |
| Image quality | 104.01 | < 0.001 | 14.12 | < 0.001 |
| VS | 60.55 | < 0.001 | 17.27 | < 0.001 |
| Visibility of small- and medium-sized vessels | 13.04 | < 0.001 | 8.49 | < 0.001 |
| Intraluminal inhomogeneity | 7.23 | < 0.001 | 4.58 | < 0.001 |
| Background noise | 20.5 | < 0.001 | 19.31 | < 0.001 |

Note.—HR=high-resolution, VS=vessel sharpness

Table S5. Results from Cumulative Link Mixed Model Analysis: Resolution Degree at 3-T Using Visibility of Small and Medium-sized Vessels.

|  | Routine | | HR | |
| --- | --- | --- | --- | --- |
| Segments | OR | *P* value | OR | *P* value |
| ICA_Ho | 8.5 | < 0.001 | 9.96 | < 0.001 |
| ICA_Ver | 7.51 | < 0.001 | 6.92 | < 0.001 |
| ICA_C4 | 7.99 | < 0.001 | 6.75 | < 0.001 |
| ICA_C5 | 13.83 | < 0.001 | 6.67 | < 0.001 |
| ICA_C6 | 15.41 | < 0.001 | 8.32 | < 0.001 |
| ICA_C7 | 9.79 | < 0.001 | 6.94 | < 0.001 |
| OA | 1.6 | 0.017 | 2.9 | < 0.001 |
| PcomA | 3.56 | < 0.001 | 1.76 | 0.021 |
| M1 | 6.2 | < 0.001 | 3.85 | < 0.001 |
| M2 | 8.32 | < 0.001 | 4.16 | < 0.001 |
| M3 | 18.68 | < 0.001 | 7.35 | < 0.001 |
| A1 | 10.52 | < 0.001 | 5.51 | < 0.001 |
| A2 | 3.42 | < 0.001 | 2.41 | < 0.001 |
| A3 | 10.94 | < 0.001 | 5.2 | < 0.001 |
| BA | 23.22 | < 0.001 | 4.21 | < 0.001 |
| SCA | 10.71 | < 0.001 | 3.91 | < 0.001 |
| P1 | 10.82 | < 0.001 | 4.02 | < 0.001 |
| P2 | 12.44 | < 0.001 | 3.75 | < 0.001 |
| P3 | 12.89 | < 0.001 | 6.55 | < 0.001 |
| VA | 18.03 | < 0.001 | 3.67 | < 0.001 |
| PICA | 6.48 | < 0.001 | 4.43 | < 0.001 |

Note.—HR=high-resolution, ICA=internal cerebral artery, ICA_Ho=ICA horizontal petrous segment, ICA_Ver=ICA vertical petrous segment, ICA_C4=ICA cavernous segment, ICA_C5=ICA clinoid segment, ICA_C6=ICA ophthalmic segment, ICA_C7=ICA communicating segment, OA=ophthalmic artery, M1-3=M1-3 segments of middle cerebral artery, A1-3=A1-3 segments of anterior cerebral artery, PcomA=posterior communicating artery, P1-3=P1-3 segments of posterior cerebral artery, BA=basilar artery, SCA=superior cerebellar artery, VA=vertebral artery, PICA=posterior inferior cerebellar artery

Table S6. Inter-Reader Agreement (Kendall's W) for Qualitative Evaluation and Visualization of Anatomical Vessel Segments with TOF-DL and TOF-Con

| Evaluation | TOF-DL | | TOF-Con | |
| --- | --- | --- | --- | --- |
|  | W | *P* value | W | *P* value |
| Overall image quality | 0.467 | <0.001 | 0.494 | <0.001 |
| VS | 0.472 | <0.001 | 0.380 | <0.001 |
| Visibility of small- and medium-sized vessels | 0.471 | <0.001 | 0.520 | <0.001 |
| Intraluminal inhomogeneity | 0.485 | <0.001 | 0.397 | <0.001 |
| Background noise | 0.376 | <0.001 | 0.397 | <0.001 |
| ICA_Ho | 0.444 | <0.001 | 0.503 | <0.001 |
| ICA_Ver | 0.461 | <0.001 | 0.481 | <0.001 |
| ICA_C4 | 0.437 | <0.001 | 0.450 | <0.001 |
| ICA_C5 | 0.463 | <0.001 | 0.504 | <0.001 |
| ICA_C6 | 0.432 | <0.001 | 0.486 | <0.001 |
| ICA_C7 | 0.447 | <0.001 | 0.480 | <0.001 |
| OA | 0.577 | <0.001 | 0.522 | <0.001 |
| M1 | 0.485 | <0.001 | 0.518 | <0.001 |
| M2 | 0.508 | <0.001 | 0.463 | <0.001 |
| M3 | 0.494 | <0.001 | 0.472 | <0.001 |
| A1 | 0.454 | <0.001 | 0.499 | <0.001 |
| A2 | 0.397 | <0.001 | 0.423 | <0.001 |
| A3 | 0.458 | <0.001 | 0.434 | <0.001 |
| PcomA | 0.394 | <0.001 | 0.405 | <0.001 |
| P1 | 0.438 | <0.001 | 0.490 | <0.001 |
| P2 | 0.442 | <0.001 | 0.501 | <0.001 |
| P3 | 0.455 | <0.001 | 0.509 | <0.001 |
| BA | 0.464 | <0.001 | 0.431 | <0.001 |
| SCA | 0.486 | <0.001 | 0.506 | <0.001 |
| VA | 0.510 | <0.001 | 0.468 | <0.001 |
| PICA | 0.513 | <0.001 | 0.500 | <0.001 |

Note.—VS=vessel sharpness, ICA=internal cerebral artery, ICA_Ho=ICA horizontal petrous segment, ICA_Ver=ICA vertical petrous segment, ICA_C4=ICA cavernous segment, ICA_C5=ICA clinoid segment, ICA_C6=ICA ophthalmic segment, ICA_C7=ICA communicating segment, OA=ophthalmic artery, M1-3=M1-3 segments of middle cerebral artery, A1-3=A1-3 segments of anterior cerebral artery, PcomA=posterior communicating artery, P1-3=P1-3 segments of posterior cerebral artery, BA=basilar artery, SCA=superior cerebellar artery, VA=vertebral artery, PICA=posterior inferior cerebellar artery

Table S7. Three Radiologists’ Performance in Detection of Aneurysms and Steno-occlusion between Two Different Imaging Protocols

| Aneurysm | | | | Steno-occlusion | | |
| --- | --- | --- | --- | --- | --- | --- |
| Reader and Metric | TOF-Con | TOF-DL | *P* value | TOF-Con | TOF-DL | *P* value |
| Reader 1 |  |  |  |  |  |  |
| Sensitivity | 65 (47, 84) | 65 (47, 84) | 1 | 81 (67, 95) | 71 (55, 87) | 0.18 |
| Specificity | 95 (90, 99) | 94 (89, 99) | 0.74 | 94 (88, 95) | 99 (96, 100) | 0.10 |
| PPV | 81 (64, 98) | 77 (60, 94) | 0.74 | 83 (70, 97) | 96 (87, 100) | 0.14 |
| NPV | 90 (83, 97) | 89 (83, 96) | 0.95 | 92 (86, 98) | 89 (83, 96) | 0.25 |
| Accuracy | 88 (80, 93) | 87 (79, 93) |  | 90 (83, 95) | 91 (84, 95) |  |
| AUC | 0.80 (0.72, 0.87) | 0.80 (0.71, 0.87) | 0.89 | 0.87 (0.79, 0.93) | 0.85 (0.77, 0.91) | 0.56 |
| Reader 2 |  |  |  |  |  |  |
| Sensitivity | 77 (61, 93) | 73 (56, 90) | 0.56 | 77 (63, 920) | 68 (51, 84) | 0.18 |
| Specificity | 94 (89, 99) | 94 (89, 99) | 1 | 81 (72, 89) | 86 (78, 94) | 0.16 |
| PPV | 80 (64, 96) | 79 (63, 95) | 0.86 | 62 (46, 77) | 66 (50, 82) | 0.46 |
| NPV | 93 (87, 98) | 92 (86, 98) | 0.56 | 90 (83, 97) | 87 (80, 94) | 0.28 |
| Accuracy | 90 (83, 95) | 89 (81, 94) |  | 80 (71, 87) | 76 (67, 84) |  |
| AUC | 0.85 (0.77, 0.92) | 0.84 (0.75, 0.90) | 0.58 | 0.79 (0.70, 0.86) | 0.77 (0.62, 0.79) | 0.58 |
| Reader 3 |  |  |  |  |  |  |
| Sensitivity | 62 (43, 80) | 69 (51, 87) | 0.32 | 74 (59, 90) | 65 (48, 81) | 0.37 |
| Specificity | 84 (76, 92) | 88 (81, 95) | 0.51 | 91 (84, 97) | 94 (88, 99) | 0.53 |
| PPV | 55 (37, 73) | 64 (47, 82) | 0.36 | 77 (62, 92) | 80 (64, 96) | 0.72 |
| NPV | 87 (80, 95) | 90 (83, 97) | 0.25 | 90 (83, 96) | 87 (80, 94) | 0.41 |
| Accuracy | 79 (70, 86) | 83 (75, 90) |  | 86 (78, 92) | 85 (77, 91) |  |
| AUC | 0.73 (0.63, 0.81) | 0.79 (0.70, 0.86) | 0.23 | 0.83 (0.74, 0.89) | 0.79 (0.70, 0.86) | 0.54 |

Note.—Data are percentages, with 95% confidence intervals in parentheses. Twenty-one patients with a history of coil embolization or clipping for intracranial aneurysm, were excluded due to severe susceptibility artifact. The weighted generalized score statistic was used to compare PPVs and NPVs of TOF-Con and TOF-DL. DL=deep learning, Con=conventional, AUC=area under the curve

^*^ Comparison of AUC of using nonparametric area under the curve comparison

Figure S1. Representative example images of quantitative measurement

(A) Representative example of ROI placement on the source image of TOF-MRA. The first circular ROI was placed at the mid-basilar artery, on an axial slice set directly below the carotid siphon, maintaining a size of 4 mm². Subsequently, a second circular ROI was drawn on the brainstem, on the same axial slice as the first ROI, maintaining a size of 200 mm².

(B) Representative example of vessel sharpness (VS) and full-width at half maximum values (FWHM). First, a linear line perpendicular to the midpoint of the right MCA was drawn on the source image. The vertical line was always drawn on the slice where the interpeduncular cistern and optic chiasm are both visible, and it was placed at the midpoint of the visible MCA. A vertical line profile curve was generated using FIJI’s “Line profile” function. Then, using the slope of the regression line for the anterior (slope_ant_) and posterior (slope_post_) vessel borders, VS was calculated by the mean of the absolute value of slope_ant_ and the absolute value of slope_post_.

*VS= mean (abs (slope_ant_) + abs (slope_post_)).*

The FWHM value was determined using the same line profile curve, measured as the width at half the maximum intensity.
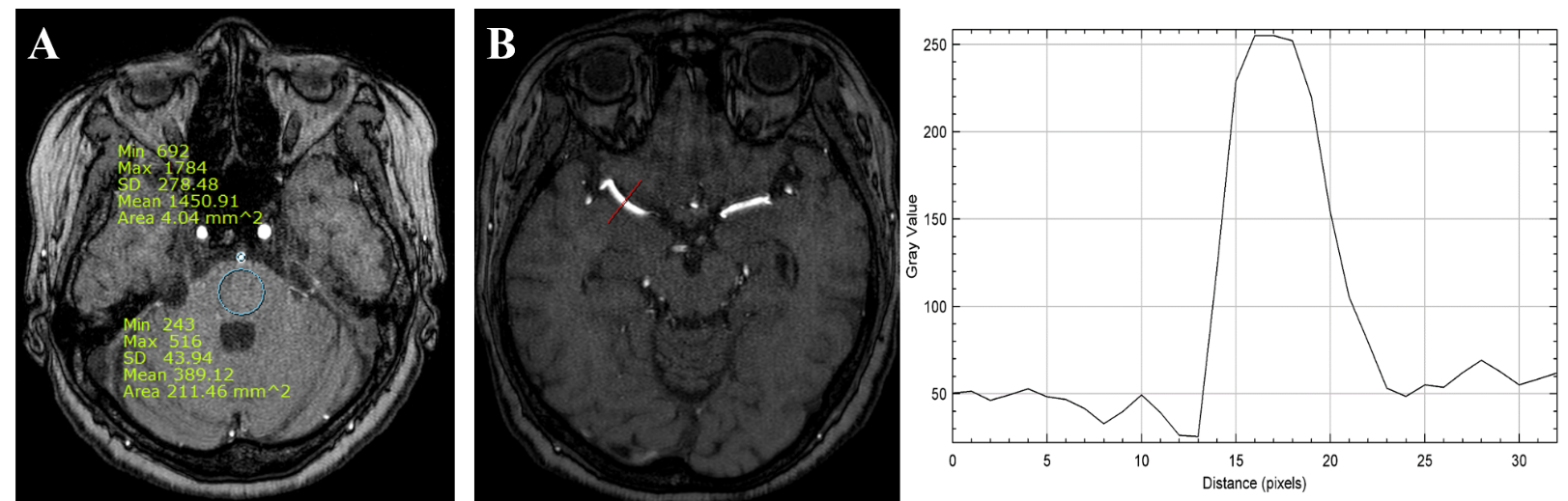


Figure S2. Imaging artifacts in TOF-MRA using a deep learning-based image enhancement program with acceleration rates of 60% or higher.

(A, B) MIP images from TOF-MRA using a deep learning-based image enhancement program (TOF-DL) with a 60% acceleration rate show the bilateral anterior cerebral arteries (ACAs) and their branches conjoined (dashed circle), which are not observed in conventional TOF-MRA. (C) A source image from conventional TOF-MRA (TOF-Con) displays prominent basal collaterals (arrows) in a patient with moyamoya disease, whereas the TOF-DL with an 80% acceleration rate diminishes these basal collaterals. These issues are likely due to decreased spatial resolution at higher acceleration rates and over-smoothing during deep learning-based reconstruction.


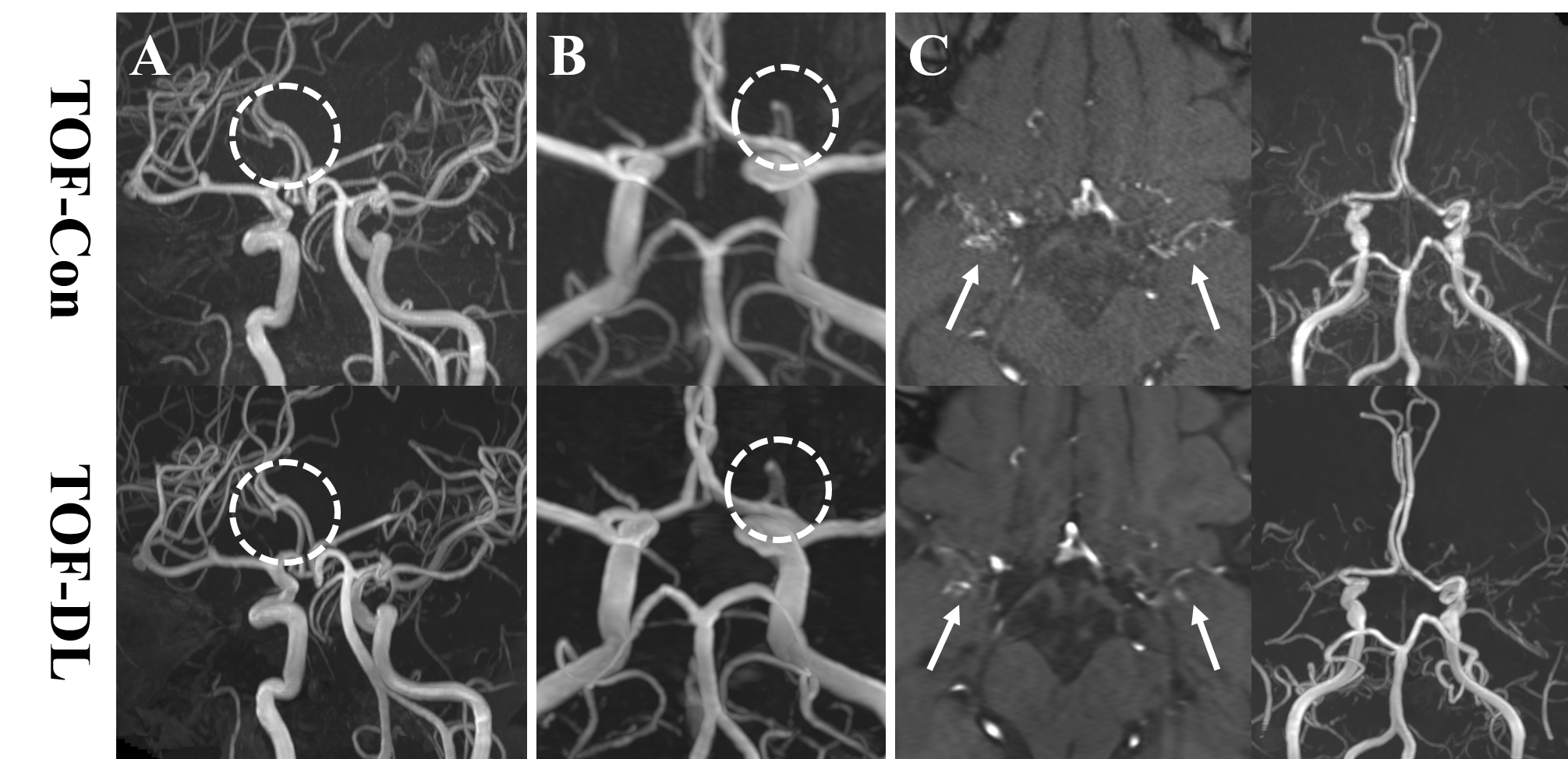

Supplement: Supplementary file 1 — Supplementary Material 1 [file 234_2025_3564_MOESM1_ESM.docx]
